# Parathyroid Hormone Induction of Cyclooxygenase-2 in Murine Osteoblasts: Role of the Calcium-Calcineurin-NFAT Pathway

**DOI:** 10.1359/jbmr.091019

**Published:** 2009-10-12

**Authors:** Hechang Huang, Daichi Chikazu, Olga S Voznesensky, Harvey R Herschman, Barbara E Kream, Hicham Drissi, Carol C Pilbeam

**Affiliations:** 1 Department of Medicine, University of Connecticut Health CenterFarmington, CT, USA; 2 Oral and Maxillofacial Surgery, University of TokyoTokyo, Japan; 3 Department of Biological Chemistry, UCLA School of MedicineLos Angeles, CA, USA; 4 Department of Orthopaedics, University of Connecticut Health CenterFarmington, CT, USA

**Keywords:** prostaglandin, MC3T3-E1, CREB, AP-1, protein kinase A

## Abstract

Murine MC3T3-E1 and MC-4 cells were stably transfected with −371/+70 bp of the murine cyclooxygenase-2 (*COX-2*) promoter fused to a luciferase reporter (Pluc371) or with Pluc371 carrying site-directed mutations. Mutations were made in (1) the cAMP response element (CRE) at −57/−52 bp, (2) the activating protein-1 (AP-1)–binding site at −69/−63 bp, (3) the nuclear factor of activated T-cells (NFAT)–binding site at −77/−73 bp, and (4) both the AP-1 and NFAT sites, which comprise a composite consensus sequence for NFAT/AP-1. Single mutation of CRE, AP-1, or NFAT sites decreased parathyroid hormone (PTH)–stimulated COX-2 promoter activity 40% to 60%, whereas joint mutation of NFAT and AP-1 abrogated the induction. On electrophoretic mobility shift analysis, PTH stimulated binding of phosphorylated CREB to an oligonucleotide spanning the CRE and binding of NFATc1, c-Fos, and c-Jun to an oligonucleotide spanning the NFAT/AP-1 composite site. Mutation of the NFAT site was less effective than mutation of the AP-1 site in competing binding to the composite element, suggesting that cooperative interactions of NFATc1 and AP-1 are more dependent on NFAT than on AP-1. Both PTH and forskolin, an activator of adenylyl cyclase, stimulated NFATc1 nuclear translocation. PTH- and forskolin-stimulated COX-2 promoter activity was inhibited 56% to 80% by calcium chelation or calcineurin inhibitors and 60% to 98% by protein kinase A (PKA) inhibitors. These results indicate an important role for the calcium-calcineurin-NFAT signaling pathway in the PTH induction of COX-2 and suggest that cross-talk between the cAMP/PKA pathway and the calcium-calcineurin-NFAT pathway may play a role in other functions of PTH in osteoblasts. © 2010 American Society for Bone and Mineral Research

## Introduction

Prostaglandins (PGs), especially PGE_2_, are abundantly produced by osteoblasts and can stimulate both formation and resorption of bone.(1) Cyclooxygenase (COX) catalyzes the committed step in the conversion of arachidonic acid, released from membrane phospholipids, to PGs. Although there are two kinds of COX, COX-1 and COX-2, the inducible enzyme, COX-2, is responsible for most PGs produced by osteoblasts.(1–3) Multiple factors can induce the expression of COX-2 in osteoblasts, including hormones, cytokines, and mechanical loading.(1)

Parathyroid hormone (PTH) is a major physiologic regulator of bone remodeling and calcium homeostasis. Although PTH stimulates both bone resorption and formation, PTH injected intermittently increases bone formation more than resorption. Recombinant human PTH(1-34) is the only anabolic therapy currently approved in the United States for treatment of osteoporosis.(4–6) PTH is a potent inducer of COX-2 in osteoblasts via the cyclic adenosine monophosphate (cAMP) protein kinase A (PKA) pathway.(7–9) Because PTH and PGE_2_ can have similar effects on bone remodeling, it is possible that some of the local effects of PTH may be mediated via the induction of COX-2 expression and PGE_2_ production.

Nuclear factor of activated T-cells (NFAT) is a family of transcription factors initially identified as regulating cytokine and early immune-response gene expression in T cells(10) and now known to be important in regulating gene expression and cell behavior in many nonimmune cells, including cardiomyocytes,(11) endothelial cells,(12,13) and glomerular mesangial cells.(14) There are five known NFAT members. NFAT5 is regulated by osmotic pressure. NFATc1 through NFATc4 are chiefly activated by the calcium-calcineurin pathway. Inactive, phosphorylated NFATs (c1–4) are located in the cytoplasm. When intracellular calcium ion concentration increases, calcineurin can be activated and dephosphorylate NFAT proteins, which then are translocated into the nucleus and become functional.(15) The activity of NFAT proteins may be modulated by interaction with transcription partners. NFAT proteins and AP-1 heterodimers (Fos/Jun) can bind cooperatively to a composite DNA site [GGA(N)9TCA] to activate gene expression.(16) NFATc1 plays an essential role in osteoclast differentiation,(17,18) and NFATc1 and NFATc2 are important in osteoblast differentiation.(19,20)

NFAT regulates COX-2 expression in several cell types. NFAT and AP-1 proteins were essential for increased COX-2 expression in T-lymphocytes in response to phorbol 12-myristate 13-acetate (PMA) and calcium ionophore.(21) NFAT also was shown to mediate the stimulation of COX-2 expression by vascular endothelial growth factor (VEGF) in endothelial cells(13) and by endothelin-1 in glomerular mesangial cells.(14)

In this study we show that PTH induces COX-2 transcription in murine osteoblastic cells via both CRE- and NFAT/AP-1–binding sites. This induction involves crosstalk between the cAMP-PKA pathway, which is thought to mediate the anabolic effects of PTH in bone, and the calcium-calcineurin-NFAT signaling pathway. Hence it is possible that some of the anabolic actions of PTH may involve the NFAT pathway.

## Materials and Methods

### Materials

The promoterless luciferase vector, the DNA constructs consisting of −371/+70 bp of the murine *COX-2* gene (Pluc371), as well as 5′ deletions of the −371/+70 bp region, fused to a luciferase reporter gene in pXp-2 vector, have been described previously.(22–24) H-89, KT5720, and GF 109203X were purchased from Biomol (Plymouth Meeting, PA, USA). The NFAT-selective inhibitor VIVIT peptide was purchased from Calbiochem (San Diego, CA, USA). The PKA agonist 6-Bnz-cAMP and the Epac agonist 8-pCPT-2'-O-Me-cAMP were purchased from Biolog Life Science Institute (San Diego, CA, USA). The retroviral vector pMSCV-GFP and the pMSCV–constitutively active NFATc1 (caNFATc1)–GFP plasmid constructs were kind gifts of Dr Neil A Clipstone (Loyola University). Synthetic bovine PTH (1-34), forskolin, phorbol myristate acetate (PMA), and other chemicals were purchased from Sigma-Aldrich (St. Louis, MO, USA).

### Cell culture

MC3T3-E1 cells, immortalized from neonatal murine calvariae, were the kind gift of Dr Yoshiyuki Hakeda (Meikai University School of Dentistry, Sakado, Saitama, Japan). The MC-4 cell line, subcloned from MC3T3-E1 cells by Dr Franceschi at the University of Michigan,(25) was purchased from the American Type Culture Collection (CRL-2593, ATCC, Manassas, VA, USA). MC3T3-E1 cells were grown in phenol red–free Dulbecco's modified Eagle's medium (DMEM, Sigma-Aldrich), and MC-4 cells were grown in α modified Eagle's medium (α-MEM, (Invitrogen, Carlsbad, CA, USA). Both media contained 10% heat-inactivated fetal calf serum (FCS, Gibco, BRL, Gaithersburg, MD, USA), penicillin (100 U/mL), and streptomycin (50 µg/mL). Cells were plated in 6 well dishes at 5000/cm^2^ and grown until confluent in a humidified atmosphere of 5% CO_2_ at 37°C. MC3T3-E1 cells were changed to serum-free medium with 1% BSA 24 hours before treatment. Treatments were pulsed into MC-4 cultures without changing the medium to avoid the COX-2 induction effects of fresh serum. Inhibitors were pulsed 1 hour before treatment with PTH or forskolin.

### Stable transfection

Stable transfections of MC3T3-E1 or MC-4 cells were performed as described previously.(26) After selection, colonies (>200) were pooled to minimize effects secondary to variable integration sites. Cells were grown in culture medium containing 200 µg/mL of G418 (Invitrogen). To maintain uniform cell phenotype, all constructs to be studied were transfected at the same time. Passage number after transfection was restricted to less than 10.

### Luciferase activity

Luciferase activity was measured in soluble cell extracts prepared with a kit from Promega (Madison, WI, USA) using an automatic injection luminometer (Berthold Lumat, Wallac, Inc., Oak Ridge, TN, USA) For each experiment, 3 wells of a 6 well dish of cells were analyzed per treatment group. Luciferase activity was measured as relative light units per second (RLU/s) and normalized to total protein measured with a bicinchoninic acid (BCA) protein assay kit (Thermo Scientific, Rockford, IL, USA). Using these normalized values, fold induction of luciferase activity was calculated as the ratio of each sample to the mean activity for the appropriate control group.

### Site-directed mutation

The template sequence for all mutations was the murine −371/+70 bp *COX-2* DNA construct. The *AP-1* sequence (5'-AGAGTCA-3') at −69/−63 bp was changed to 5'-AGAGTtg-3' (*muAP-1*) as described previously.(26) The *CRE* (5'-CGTCA-3') at −56/−52 bp was changed to 5'-atTCA-3' (*muCRE*) as described previously.(26,27) The *NFAT* sequence (5'-GGAAA-3') at −77/−73 bp was changed to 5'-ttAAA-3' (*muNFAT*) using the QuickChange Site-Directed Mutagenesis Kit from Stratagene (La Jolla, CA, USA) following the manufacturer's directions. After introduction of deletions or mutations, the *COX-2* 5'-flanking region was sequenced (Automated DNA Sequence Facility, University of Connecticut Health Center, Farmington, CT, USA). Mutated oligonucleotides were used as unlabeled competitors on electrophoretic mobility gel-shift assay (EMSA) to confirm that binding to the mutated sequence did not occur.

### Electrophoretic mobility gel-shift assay (EMSA)

Cells were washed with PBS and collected by centrifugation, and nuclear extracts were obtained using a kit (BioVision Research Prodicts, Mountain View, CA, USA). Single-stranded oligonucleotides (Integrated DNA Technologies, Coralville, IA, USA) were annealed with complementary oligonucleotides, and the resulting double-stranded DNAs were end labeled with γ^32^P-ATP (PerkinElmer, Waltham, MA, USA) using T_4_ kinase (Invitrogen). The 6 µg of nuclear extract was incubated in 20 µL binding-reaction mixture [10 mM Tris HCl, pH 7.5, 1 mM DTT, 1 mM EDTA, 5% glycerol, and 2 µg poly dI-dC (Amersham Biosciences, Piscataway, NJ, USA)] with 50,000 cpm of purified labeled probe. Nondenaturing acrylamide gel (5%) electrophoresis was performed for 2 hours. Competitors (50 to 300 M excess) or supershifting antibodies (4 µg) were added to the binding mixture 30 minutes before addition of the probe, and incubation was continued for 30 minutes at 4°C. Antibodies to NFATc1-c4, c-Fos or c-Jun, and phosphorylated CREB were obtained from Santa Cruz Biotechnology (Santa Cruz, CA, USA). Normal mouse and rabbit IgGs were purchased from Millipore (Billerica, MA, USA). Dried gels were exposed to X-ray film or phosphor image plate.

### Western blot analysis

Nuclear and cytosolic extracts were obtained with a fractionation kit from BioVision. Protein concentrations were measured by BCA assay (Thermo Scientific). Equal amounts (25 or 40 µg) of protein were used for 10% SDS-PAGE and transferred to nitrocellulose membrane (Bio-Rad Lab, Helcules, CA, USA). Membranes were washed with Tris-buffered saline (TBS, pH 7.6), blocked with 5% wt/vol nonfat dry milk in 1 × TBS containing 0.05% Tween 20 (TBST) overnight at 4°C, and incubated with primary antibody (Santa Cruz) at 1:200 dilution. After washing with TBST, membranes were incubated with horseradish peroxidase–conjugated secondary antibody at 1:1,000 dilution. The signal was detected with LumiGLO chemiluminescent reagent (Cell Signaling Technology, Danvers, MA, USA).

### Real-time (quantitative) PCR analysis

Gene expression studies were done using TaqMan fluorogenic probes. Total RNA was extracted with Trizol (Invitrogen) following the manufacturer's instructions. Then 5 µg of total RNA was converted to cDNA using the high-capacity cDNA Archive Kit (Applied Biosystems, Foster City, CA, USA) according to the manufacturer's directions. Real-time PCR for *COX-2* expression was performed in 96 well plates using the Assays-on-Demand gene expression system (Applied Biosystems) with TaqMan fluorogenic probes and glyceraldehyde 3-phosphate dehydrogenase (*GAPDH*) as the endogenous control. Each sample was amplified in duplicate. The PCR reaction mixture (20 mL/well including 2 × TaqMan Universal PCR Master Mix, 20 × Assays-on-Demand Gene Expression assay mix, and 100 ng of cDNA) was run in the ABI Prism 7300 Sequence machine universal thermal cycling parameters. The standard-curve method was used for data analysis.

### Chromatin immunoprecipitation (ChIP) assay

ChIP assay was performed using the EZ-CHIP Kit from Millipore following the manufacturer's instructions. MC-4 cells were grown to confluency, treated for 1 hour with PTH (10 nM) or vehicle, cross-linked with formaldehyde, lysed, sonicated with six sets of 20-second pulses, and soluble chromatin harvested. Supernatant was precleared with protein G agarose and purified by centrifugation. Immunoprecipitation was performed by incubating the samples overnight at 4°C with 2 µg of mouse NFATc1 antibody (Santa Cruz) or 1 µg anti-RNA polymerase antibody (positive control) or 1 µg of mouse IgG antibody (negative control), followed by incubation with protein G agarose for 1 hour at 4°C to collect antibody-antigen-DNA complexes. Protein-DNA complexes were eluted from the agarose and reverse cross-linked to free the DNA, which was then purified using spin columns. The DNA solution also was used as the template for SYBR Green quantitative polymerase chain reaction (PCR). Specific primers encompassing the NFAT binding sites on the *COX-2* promoter (from nucleotide −135 to −4 bp) were designed using the NCBI/Primer-BLAST program (http://www.ncbi.nlm.nih.gov/BLAST). Experimentally validated murine primer pairs for actin (ID 6671509a1) were found in PrimerBank (http://pga.mgh.harvard.edu/primerbank). Primers were made by Integrated DNA Technologies. The PCR reaction mixture for SYBR Green assays contained 2× SYBR Green PCR Master Mix (Applied Biosystems) forward and reverse primer mix at final concentration for each primer of 200 nM and 2 µL of DNA solution. The reaction was run in triplicate for each sample under a common PCR thermal profile: 50°C for 2 minutes, 95°C for 10 minutes, 95°C for 15 seconds, 60°C for 30 seconds, and 72°C for 30 seconds. PCR products were analyzed by gel electrophoresis (37 cycles) and by real-time PCR (40 cycles). For real-time PCR, we started with equal amounts of chromatin for each sample, and the *C*_*t*_ values for each sample were calculated without normalization. The ChIP assay was done in duplicate.

### Statistical analysis

Means of groups were compared by one-way analysis of variance (ANOVA) using SigmaStat for Windows, Version 2.03 (San Rafael, CA, USA). Significance of differences was determined by post hoc testing using Bonferonni *t* test or the Tukey test.

## Results

### Site-directed mutation of CRE, AP-1, and NFAT elements

Studies were done in both preosteoblastic MC3T3-E1 cells and MC-4 cells. MC-4 cells were subcloned from MC3T3-E1 cells because of concerns that MC3T3-E1 cells had become a mixed population of phenotypes after years of being carried by various laboratories and were shown to have high differentiation and mineralization potential.(25) All studies were done in stably transfected cells grown for 6 to 7 days after plating. PTH has been shown to rapidly induce transcription and expression of COX-2 in MC3T3-E1 cells.(8,9) In both MC3T3-E1 and MC-4 cells stably transfected with Pluc371, PTH-stimulated luciferase activity peaked at 2 to 3 hours and returned to baseline by 8 hours (1*A, B*). We used the 3 hour time point for all further studies of luciferase activity.

**Fig. 1:**
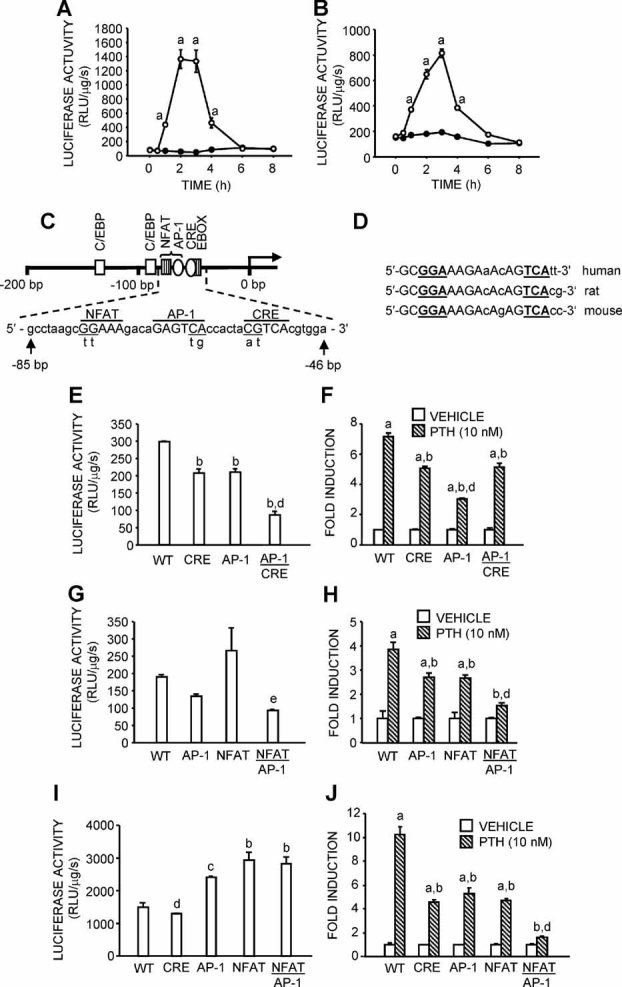
Induction of murine *COX-2* promoter activity by PTH in MC3T3-E1 and MC-4 cells is dependent on the *CRE*, *AP-1*, and *NFAT* sites. Cells stably transfected with wild-type (WT) Pluc371 or Pluc371 with 2 bp mutations in the *CRE*, *AP-1*, and *NFAT* sites were treated with vehicle or PTH (10 nM). Unless otherwise specified, cells were treated for 3 hours. Luciferase activity was calculated as relative light units (RLU)/s and normalized to total protein. Using the normalized values, fold induction of luciferase activity was calculated as the ratio of each sample activity to the mean activity for the appropriate control group. Time course for the PTH induction of *COX-2* promoter luciferase reporter activity in (*A*) MC3T3-E1 cells and in (*B*) MC-4 cells carrying Pluc371. (*C*) Location in the murine *COX-2* 5′-flanking region of the mutated cis-acting elements. (*D*) Comparison of nucleotide sequences in the −80/−60 bp *COX-2* 5'-flanking regions of the human, rat and mouse genes. (*E*) Effects of mutating the *CRE*, *AP-1*, or both *CRE* and *AP-1* sites in MC3T3-E1 cells on basal luciferase activity and (*F*) PTH-stimulated fold increase in luciferase activity. (*G*) Effects of mutating the *AP-1*, *NFAT*, or both *AP-1* and *NFAT* sites in MC3T3-E1 cells on basal luciferase activity, and (*H*) PTH-stimulated fold increase in luciferase activity. (*I*) Effects of mutating the *CRE*, *AP-1*, and *NFAT* sites in MC-4 cells on basal luciferase activity, and (*J*) PTH-stimulated fold increase in luciferase activity. Bars or symbols are means ± SEM for *n* = 3 wells of cells. ^a^Significant effect of PTH, *p* < .01. ^b^Significantly different from WT, *p* < .01. ^c^*p* < .05. ^d^Significantly different from all other mutations, *p* < .01. ^e^Significantly different from the single *NFAT* mutation, *p* < .05.

We made mutations in the cAMP response element (*CRE*) that is important for the regulation of COX-2 expression by multiple agents,(27) an *AP-1*-binding site that partially mediates effects of PMA and serum on COX-2,(2) and an *NFAT* consensus sequence(21) (see 1*C*). The sequence 5′-GGAAAGACAGAGTCA-3 spanning the *NFAT* and *AP-1* sites is identical to an *NFAT*/*AP-1* composite consensus sequence GGA(N)9TCA(12) and is conserved among the human, rat, and mouse genomic sequences(21) (see 1*D*). NFAT and AP-1 (Fos/Jun) proteins have been shown to interact cooperatively to mediate transcription.(12,28,29) We compared mutated constructs only in cells transfected simultaneously with those constructs.

Mutations of the *CRE*, the *AP-1* site, and both sites were compared in MC3T3-E1 cells. A representative experiment is shown in 1*E, F*. PTH (10 nM) significantly induced luciferase activity 7.2-fold in one experiment (see 1*F*) and 3.3- and 5.3-fold in two other experiments (data not shown). *AP-1* and *CRE* mutations significantly decreased the PTH-stimulated fold activity on average 60% and 50%, respectively. There was no additional reduction in the response to PTH with the combination of *AP-1* and *CRE* mutations in any of the three experiments. Although the basal activity was variable among experiments, the joint *AP-1* and *CRE* mutation consistently reduced basal luciferase activity (on average 76%) more than the individual mutations (40% to 46%).

Mutations of the *AP-1* site, the *NFAT* site, and both sites also were compared in MC3T3-E1 cells. A representative experiment is shown in 1*G, H*. PTH significantly induced luciferase activity 5.6-fold (see 1*H*) in one experiment and 3.8- and 8.9-fold in the other two experiments (data not shown). Single mutation of the *AP-1* site significantly reduced the PTH stimulated fold induction in two of the three experiments by 39%. Single mutation of the *NFAT* site significantly reduced the fold induction of PTH in all three experiments by 18% to 40%. In contrast, the PTH-stimulated fold increase was decreased 82%, 94%, and 96% by the joint *AP-1* and *NFAT* mutations. The mutations produced no consistent differences in basal activity.

Mutations of the *CRE*, the *AP-1* site, the *NFAT* site, and both the *AP-1* and *NFAT* sites were compared in MC-4 cells. A representative experiment is shown in 1*I, J*. In this experiment, PTH induced a 10-fold increase in luciferase activity that was reduced 61%, 54%, 64%, and 93% by the *CRE*, *AP-1*, *NFAT*, and joint *AP-1* and *NFAT* mutations, respectively. In a second experiment, PTH induced a 5.8-fold increase in luciferase activity that was significantly reduced 50%, 48%, 38%, and 98%, respectively, by the *CRE*, *AP-1*, *NFAT*, and joint *AP-1* and *NFAT* mutations, respectively (data not shown). In a third experiment, PTH induced a 7-fold increase in luciferase activity that was significantly reduced 48%, 50%, and 94% by the *AP-1*, *NFAT*, and combined mutations, respectively (data not shown).

In the six independent experiments in MC3T3-E1 and MC-4 cells, there was no statistically significant induction of luciferase activity by PTH in any group with joint *AP-1* and *NFAT* mutations, suggesting that the composite *NFAT*/*AP-1* element plays a critical role in the PTH induction of *COX-2* promoter activity. As noted earlier, basal activity was quite variable even in repeated assays on cells carrying the same construct, suggesting that despite pooling of multiple clones for the transfection, some phenotypic differences occurred during the selection process or during passage. The joint *NFAT*/*AP-1* mutation increased basal activity significantly in three experiments, reduced basal activity significantly in two experiments, and had no effect on basal activity in the remaining experiment. Hence the abrogation of PTH-stimulated luciferase activity by the combined *AP-1* and *NFAT* mutations was independent of effects on basal activity.

### Binding of nuclear proteins to CRE, AP-1, and NFAT elements

We performed EMSA on MC-4 cells treated with vehicle or PTH (10 nM) for 1 hour. There was constitutive binding of a ^32^P-labeled COX-2 oligonucleotide (−63/−39 bp) spanning the *CRE* site on EMSA (2*A*, band *a* in lanes 2, 3, 8, and 9), with increased binding seen on some gels after PTH treatment. This binding was competed by unlabeled oligonucleotide (lanes 4 and 5) but not by unlabeled oligonucleotide with mutation of the *CRE* (MUT; lanes 6 and 7). Antibody to phosphorylated CREB (pCREB) supershifted a band (lane 11, band *b*) in PTH-treated nuclear extracts but not in vehicle-treated samples (lane 10). No supershift was observed in extracts incubated with nonspecific IgG (lanes 12 and 13).

**Fig. 2:**
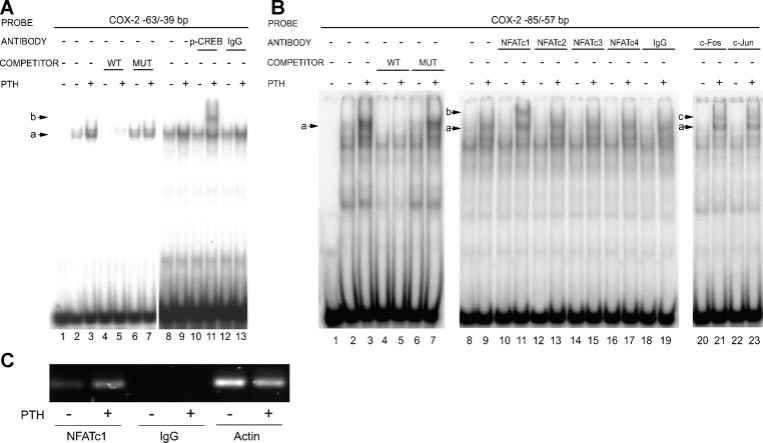
PTH induces binding of nuclear proteins to the *CRE*, *NFAT*, and *AP-1* elements. EMSA and ChIP analyses were performed on MC-4 cells treated with PTH (10 nM) for 1 hour. (*A*) Binding of nuclear proteins extracted from MC-4 cells to a ^32^P-labeled COX-2 oligonucleotide (−63/−39 bp) spanning the *CRE* (−57/−52 bp). Binding (*arrow a*, lanes 2, 3, 8, and 9) was competed by unlabeled wild-type (WT) oligonucleotide (lanes 4 and 5) but not by oligonucleotide with 2 bp mutation of the *CRE* (lanes 6 and 7) and supershifed by antibody to phoshorylated CREB (pCREB) (*arrow b*, lane 11) but not by IgG (lane 13). Lane 1: Free probe. (*B*) Binding of nuclear proteins extracted from MC-4 cells to a ^32^P-labeled COX-2 oligonucleotide (−85/−57 bp) spanning the composite *NFAT*/*AP-1* element (−77/−63 bp). PTH-induced binding (*arrow a*, lanes 2 and 3) was competed by unlabeled WT oligonucleotide (lanes 4 and 5) but not by oligonucleotide with mutation of the *AP-1* and *NFAT* sites (lanes 6 and 7). Supershift was seen with antibody to NFATc1 (*arrow b*, lane 11) but not with antibodies to NFATc2–4 or IgG (lanes 13, 15, 17, and 19). Supershift also was seen with antibodies to c-Fos (*arrow c*, lane 21) and c-Jun (lane 23). Lane 1: Free probe. (*C*) ChIP assay for binding of NFATc1 to the native *COX-2* promoter. Soluble chromatin extracts from MC-4 cells treated with vehicle or PTH were incubated with anti-NFATc1 antibody, nonimmune IgG, or no antibody (input). Immunoprecipitated DNA was analyzed by PCR using primers spanning −135/−4 bp of the *COX-2* promoter region. PCR products were run on 2% agarose gel and stained with ethidium bromide.

PTH induced binding of a ^32^P-labeled oligonucleotide (−85 to −57 bp) that spanned the *NFAT* and *AP-1* composite site (see 2*B*, lane 3, band *a*). This binding was competed by unlabeled oligonucleotide (lanes 4 and 5) but not by unlabeled oligonucleotide with mutations in both *AP-1* and *NFAT* sites (lanes 6 and 7). Antibody to NFATc1 supershifted a band in PTH-treated extracts (lane 11, band *b*). In contrast, antibodies to NFATc2 (lanes 12 and 13), NFATc3 (lanes 14 and 15), and NFATc4 (lanes 16 and 17) did not alter the position of this band. Antibodies to c-Fos and c-Jun also gave rise to retarded bands (lanes 21 and 23, respectively). No supershifted band was seen with IgG (lane 19). Similar results were seen with EMSA in MC3T3-E1 cells (data not shown).

To demonstrate binding of NFATc1 to native chromatin in MC-4 cells, we performed ChIP using primers spanning −135/−4 bp of the *COX-2* promoter region. PCR products run on agarose gel and stained with ethidium bromide are shown in 2*C*. Products also were analyzed by real-time PCR. The difference in *C*_*t*_ values between control and PTH-treated samples was 4.8 in one experiment and 3.9 in a second experiment, indicating an increase in PTH-stimulated NFATc1 binding of about 20-fold. We cannot rule out binding to several other potential *NFAT* elements in the −135/−4 bp promoter sequence, which spans two additional GGAAA sequences at −90/−86 and −111/−107 bp. However, mutation of either of these sites in combination with mutation of the *AP-1* site studied in stably transfected MC-4 cells resulted in no greater decrease in PTH-stimulated luciferase activity than the *AP-1* mutation alone (data not shown).

**Fig. 3:**
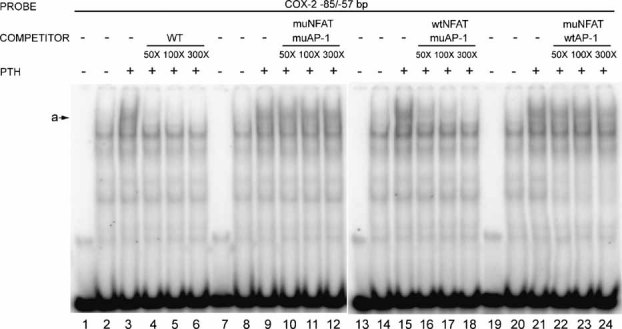
Mutating the *NFAT* element has a greater effect on nuclear protein binding to the composite *NFAT*/*AP-1* sequence than mutating the *AP-1* element. EMSA was performed on nuclear extracts from MC-4 cells treated with PTH (10 nM) for 1 hour using a ^32^P-labeled COX-2 oligonucleotide (−85/−57 bp) spanning the composite *NFAT*/*AP-1* element (−77/−63 bp). Unlabeled wild-type oligonucleotide (WT, lanes 4 to 6) or unlabeled oligonucleotide with mutations in both *AP-1* and *NFAT* (lanes 10 to 12), mutations in *AP-*1 only (*muAP-1*, lanes 16 to 18) or *NFAT* only (*muNFAT*, lanes 22 to 24) were used at 50, 100, and 300 M excess. Lanes 1, 7, 13, and 19: Free probe.

To study the cooperative binding of NFAT and AP-1 proteins to the composite *NFAT*/*AP-1* element, we compared the ability of oligonucleotides carrying the single *AP-1* mutation (*muAP-1*) or *NFAT* mutation (*muNFAT*) to compete the binding of nuclear proteins to the *NFAT*/*AP-1* element, as described previously(29) (Fig. 3). Unlabeled oligonucleotide with *muAP-1* (lanes 16 to 18) was almost as effective as the wild-type oligonucleotide (lanes 4 to 6) at competing for the formation of the protein-DNA complexes and much more effective than the oligonucleotide with *muNFAT* (lanes 22 to 24). These observations suggest that interactions at the composite *NFAT*/*AP-1* element are more dependent on *NFAT* than on *AP-1*.

### Involvement of the cAMP-PKA pathway

PTH exerts its effect on bone metabolism by binding to the G protein–coupled PTH/PTH-related peptide receptor.(30,31) PTH binding to this receptor stimulates adenylate cyclase through Gα_s_ protein, thereby increasing cAMP and activating PKA. Although PTH is thought to mediate most of its biologic effects in osteoblastic cells via cAMP-PKA signaling, PTH also can activate the protein kinase C (PKC) pathway.(32,33) H-89, a PKA inhibitor, inhibits PTH induction of *COX-2* transcription and *COX-2* mRNA and protein expression in MC3T3-E1 cells.(8,9) However, H-89 also strongly inhibits the induction of *COX-2* by PMA, a PKC agonist.(34–36) To rule out involvement of the PKC pathway, we examined effects of a specific PKC inhibitor GF 109203X on the induction of luciferase activity in MC-4 cells in response to PMA (1 µM) and forskolin (1 µM), an adenylate cyclase agonist. As reported previously,(34–36) GF 109203X inhibited the PMA induction (2.6-fold) of luciferase activity by 80% (data not shown), with no effect on the forskolin induction (7.1-fold) of luciferase activity (data not shown). GF 109203X had no effect on the PTH induction of luciferase activity (4*A*). Consistent with earlier studies, H-89 abrogated the PTH induction of luciferase activity (4*B*). A more specific PKA inhibitor, KT 5720,(37) significantly inhibited the forskolin induction (7.9-fold) of luciferase activity by 64% (data not shown) and the PTH induction of luciferase by 60% (see 4*B*).

**Fig. 4:**
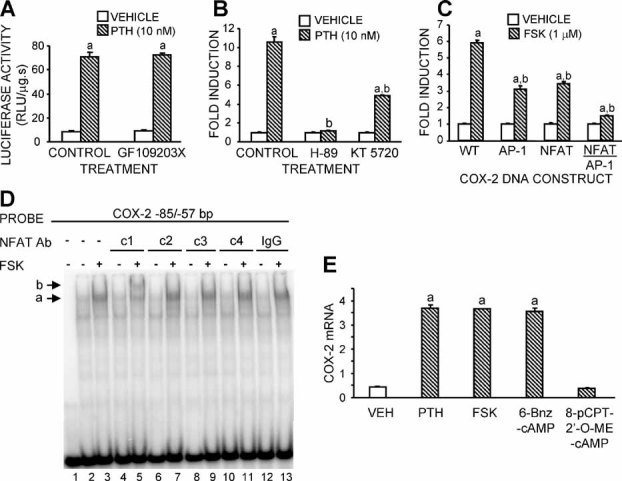
PTH induction of *COX-2* promoter activity depends on the cAMP-PKA pathway. (*A*, *B*) Effects of the specific PKC inhibitor GF109203X (0.6 µM) and the PKA inhibitors H-89 (30 µM) and KT 5720 (10 µM) on the luciferase activity or fold induction in response to PTH. MC-4 cells stably transfected with Pluc371 were treated with vehicle, PTH, or forskolin (FSK). Inhibitors or vehicle (Control) were added 1 hour before PTH. (*C*) Effects of mutating the *AP-1* site, the *NFAT* site, and both on the response to forskolin. MC-4 cells stably transfected with wild-type (WT) Pluc371 or Pluc371 with mutations in the *AP-1* and *NFAT* sites were treated for 3 hours with vehicle or forskolin (FSK). Luciferase activity was calculated as relative light units (RLU)/s and normalized to total protein. Using the normalized values, fold induction of luciferase activity was calculated as the ratio of each sample to the mean activity for the appropriate control group. Bars are means ± SEM for *n* = 3 replicates. ^a^Significant effect of PTH or forskolin, *p* < .01. ^b^Significant effect of inhibitor or mutation, *p* < .01. (*D*) EMSA of forskolin-induced nuclear binding to a ^32^P-labeled COX-2 oligonucleotide (−85/−57 bp) spanning the composite *NFAT*/*AP-1* element (−77/−63 bp). MC-4 cells were treated with forskolin (FSK, 1 µM) for 1 hour. Forskolin-induced binding (*arrow a*, lane 3) was supershifted by antibody to NFATc1 (*arrow b*, lane 5) but not by antibodies to NFATc2–4 (lanes 7, 9, and 11) or IgG (lane 13). Lane 1: Free probe. (*E*) Effects of specific PKA and Epac agonists on *COX-2* mRNA expression, measured by real-time RT-PCR. MC-4 cells were treated with vehicle (VEH), PTH (10 nM), forskolin (FSK, 1 µM), 6-Bnz-cAMP (1 mM), and 8-pCPT-2′-O-Me-cAMP (1 mM) for 1 hour. Bars are means ± SEM for *n* = 3 replicates. ^a^Significant effect of agonist, *p* < .01.

Similar to the effects seen with PTH, the forskolin induction of luciferase activity in stably transfected MC-4 cells was inhibited 50% to 60% by mutation of the *AP-1* or *NFAT* site and abrogated by joint mutation of these two sites (see 4*C*). On EMSA, forskolin treatment induced binding (band *a* in lane 3) that was supershifted by antibody to NFATc1 (band *b* in lane 5) but not by antibodies to other members of the NFAT family (see 4*D*).

Exchange protein directly activated by cAMP (Epac) is another target of cAMP that mediates effects of cAMP independent of the PKA pathway. Epac is expressed in MC-4 cells and was shown to mediate effects of PTH on cell proliferation.(38) We compared treatment with high doses (1 mM) of the specific PKA agonist 6-Bnz-cAMP and the specific Epac agonist 8-pCPT-2′-O-Me-cAMP on *COX-2* mRNA expression in MC-4 cells.(39) 6-Bnz-cAMP induced *COX-2* mRNA more than 8-fold, similar to induction by PTH or forskolin, whereas 8-pCPT-2′-O-Me-cAMP had no effect on *COX-2* message (see 4*E*).

### Regulation of calcium-calcineurin-NFAT signaling by the cAMP-PKA pathway

Increased concentration of intracellular calcium activates calcineurin, and calcineurin activates NFATc1 protein in the cytoplasm by dephosphorylation.(15) Dephosphorylated NFAT1c translocates to the nucleus. Consequently, cellular localization of NFAT1c is an indication of its activation status. We examined by Western blot the distribution of NFAT proteins in the cytoplasm and nucleus in MC-4 cells treated with vehicle or PTH. Nuclear concentration of NFATc1 was increased by PTH (5*A*). Forskolin also stimulated nuclear translocation of NFATc1, but PMA did not (5*B*).

**Fig. 5:**
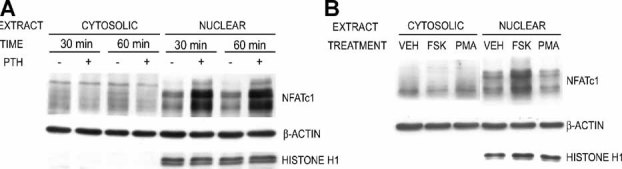
*NFATc1* nuclear translocation is induced by PTH and forskolin but not by PMA in MC-4 cells. MC-4 cells were treated for 30 to 60 minutes. Factionated nuclear and cytoplasmic proteins were extracted as described in “Materials and Methods.” (*A*) *NFATc1* nuclear translocation induced by treatment with PTH (10 nM). (*B*) *NFATc1* nuclear translocation induced by 1 hour of treatment with vehicle (VEH), forskolin (FSK, 1 µM), or PMA (1 µM). β-actin and histone H_1_ are shown to assess loading on gel.

PTH-stimulated *COX-2* mRNA expression in MC-4 cells was inhibited inhibited 88% by the calcium chelator BAPTA-AM and 58% and 65%, respectively, by the calcineurin inhibitors FK506 and cyclosporin A (CsA) (6*A*). PTH-stimulated Pluc371 activity in MC-4 cells was inhibited 99% by BAPTA-AM and 56% to 60% by the calcineurin inhibitors FK506, CsA, and VIVIT in the experiment shown in 6*B*. In a second experiment, PTH-stimulated Pluc371 activity was inhibited 81% by BAPTA-AM and 76% to 81% by FK506 and CsA (*p* < .01; data not shown). Forskolin-stimulated Pluc371 activity was inhibited 76% by BAPTA-AM and 64% to 65% by FK506 and CsA (see 6*C*). These data indicate that PTH can stimulate *COX-2* mRNA and promoter activity via crosstalk between the cAMP-PKA and calcineurin-NFAT signaling pathways.

**Fig. 6:**
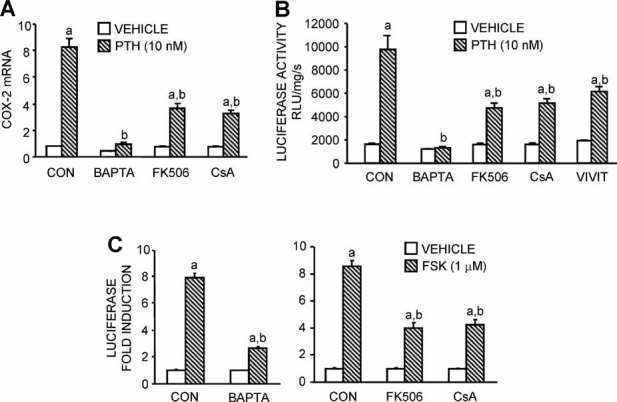
PTH induction of *COX-2* expression and promoter activity depends on calcium-calcineurin signaling. MC-4 cells stably transfected with Pluc371 were treated with vehicle, PTH (10 nM), or forskolin (FSK, 1 µM) for 1 hour (mRNA) or 3 hours (luciferase activity). BAPTA-AM (BAPTA, 15 µM), FK506 (10 µg/mL), cyclosporin A (CsA, 10 µg/mL), or VIVIT (1 µM) were added 1 hour before PTH or forskolin. Vehicle (CON) for the inhibitors, DMSO, also was added 1 hour before PTH or forskolin. Luciferase activity was normalized to total protein and used to calculate the fold induction as the ratio of treatment to control. (*A*) Effects of the calcium chelator BAPTA-AM and the calcineurin inhibitors FK506 and CsA on PTH-induced *COX-2* mRNA expression measured by real-time RT-PCR. (*B*) Effect of BAPTA-AM and the calcineurin inhibitors FK506, CsA, and VIVIT on the PTH induction of luciferase activity. (*C*) Effect of BAPTA-AM, FK506, and CsA on the forskolin induction of luciferase activity. Bars are means ± SEM for *n* = 3 wells of cells. ^a^Significant effect of agonist, *p* < .01. ^b^Significant effect of inhibitor, *p* < .01.

### Enhancement of COX-2 mRNA expression by NFATc1 overexpression

We stably transfected MC-4 cells with a constitutively active mutant form of *NFATc1* (*caNFATc1*) or its control vector (pMSCV-GFP).(40) Nuclear expression of NFATc1 protein was increased in cells stably transfected with *caNFATc1* compared with control cells (7*A*). Basal and PTH-induced *COX-2* mRNA expression was increased 3.3- and 1.6-fold, respectively, in the *caNFATc1* expressing cells compared with control cells (7*B*).

**Fig. 7:**
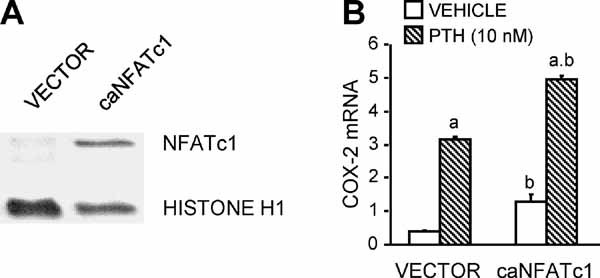
NFATc1 overexpression enhances basal COX-2 expression and induction by PTH. MC-4 cells stably transfected with pMSCV-GFP (vector) or pMSCV-caNFATc1-GFP plasmid constructs were treated with vehicle or PTH (10 nM) for 1 hour. (*A*) Nuclear protein extraction and Western blot were performed as described in “Materials and Methods.” (*B*) *COX-2* mRNA levels measured by real-time RT-PCR. Bars are means ± SEM for *n* = 3 wells of cells. ^a^Significant effect of PTH, *p* < .01. ^b^Significant effect of caNFATc1 overexpression, *p* < .01.

## Discussion

Our results are consistent with earlier studies that reported the PTH induction of *COX-2* expression and promoter activity to be mediated via the cAMP-PKA pathway.(8,9) As expected, the *CRE*, which is involved in the induction of *COX-2* promoter activity by multiple factors,(26,27,41) was found to play a significant role in the PTH induction of *COX-2* promoter activity. PTH increased binding of phosphorylated CREB-1 to the *CRE* on EMSA, and a 2-bp mutation in the *CRE* reduced PTH-stimulated *COX-2* promoter-luciferase activity 50% to 60%.

Transcriptional regulation of *COX-2* by most factors appears to involve multiple cis-acting sites.(1,42) Along with the *CRE*, the *AP-1*-binding element at −69/−63 bp has been shown to mediate induction of *COX-2* by serum and PMA.(26) PTH induced c-Fos and c-Jun binding to the oligonucleotide spanning the *AP-1*-binding site on EMSA, and a 2-bp mutation of this *AP-1* element reduced PTH-stimulated luciferase activity 40% to 60%. A role for AP-1 factors in mediating PTH effects has been found in several other systems. For example, PTH can induce collagenase expression(43,44) and c-Fos expression(45) via AP-1 factors.

The reduction in PTH stimulated *COX-2* promoter activity by a 2 bp mutation of the *NFAT* element alone ranged from 18% to 64%. The joint mutation of the *AP-1* and *NFAT* elements, which form a consensus *NFAT*/*AP-1* composite binding sequence, consistently abrogated the PTH induction of *COX-2* promoter activity. EMSA showed that PTH stimulated NFATc1 but not NFATc2–4 binding to a sequence spanning the composite site and that NFAT played a dominant role in the cooperative binding of NFAT and AP-1 proteins to the composite site. Binding of NFAT and AP-1 proteins to a composite *NFAT*/*AP-1* site is reported to be important in the regulation of expression of cytokine and surface receptor genes in immunocytes(16,46) and in the induction of *COX-2* by PMA and calcium ionophore in human T-lymphocytes(21) and by HIV-Tat in glial cells.(47) Our data indicate that this composite *NFAT*/*AP-1* element is also important for *COX-2* induction by PTH in osteoblasts and that constitutively active NFATc1 can enhance the basal and PTH-stimulated expression of COX-2 in osteoblast cultures.

Although regulation of NFAT activity has been shown to be involved in mediating *COX-2* transcription by a variety of agents,(13,14,21,47,48) this is the first study to report regulation by an agent thought to act largely via the cAMP-PKA pathway. Previous studies on the relationship between the cAMP-PKA pathway and the calcium-calcineurin-NFAT pathway have reported differing results. PKA had inhibitory effects on NFAT activity in T-lymphocytes and mouse lymphoma EL-4 cells(49–51) but activated NFAT proteins in pancreatic islet β cells.(52,53) In this study, the adenylate cyclase agonist forskolin mimicked the effects of PTH, stimulating binding of nuclear proteins to the *NFAT*/*AP-1* composite element and activating *NFATc1* nuclear translocation. Inhibitors of PKA inhibited the stimulation of *COX-2* promoter activity by PTH and forskolin similarly. Calcium chelation as well as calcineurin inhibitors significantly blunted effects of both PTH and forskolin on *COX-2* promoter activity. PTH has been shown to activate ERK phosphorylation via Epac in MC-4 cells.(38) Although we cannot rule out a role for Epac, our data strongly suggest that PTH activates the calcium-calcineurin-NFAT pathway via cAMP-PKA signaling.

One possible mechanism for the association of cAMP-PKA and calcium-calcineurin activity in osteoblasts might be the family of scaffold proteins called *A kinase-anchoring proteins* (AKAPs). In a variety of cells, including T-lymphocyte, adipocytes, cardiomyocytes, and myocytes, PKA can form a signal-transduction complex with AKAPs located in different subcellular compartments through interactions with lipids or other proteins.(54) Our preliminary studies show expression of AKAP79 and AKAP18 proteins in MC-4 cells (data not shown). Some studies report that calcineurin can be a part of the AKAP complex and that PKA activated by cAMP can sensitize calcium channels on the cell compartment membrane, increasing the concentration of calcium ions in the cytosol, which, in turn, activates calcineurin and NFAT proteins.(55–57)

Although NFAT proteins were identified originally as controlling cytokine and early immune-response gene expression in T cells,(10) they are now found to regulate gene expression in a variety of cells.(11–14) NFATc1 plays a pivotal role in osteoclast differentiation.(17,18) NFAT proteins are also likely to be important in osteoblast differentiation and function. Although one study showed that overexpression of NFATc1 in vitro inhibited osteoblast differentiation,(58) other studies have shown that NFATc1 deficiency suppressed osteoblast differentiation in vitro(19); NFATc1 or NFATc2 deficiency caused a defect in bone formation in vivo(19,20); and NFATc1 overexpression in vivo resulted in increased osteoblast proliferation and high bone mass.(20)

PTH stimulates bone formation and resorption by acting on cells of the osteoblastic lineage to increase both their differentiation into mature matrix-producing cells and their essential support for the differentiation of osteoclasts. The results of our study show that PTH can activate the calcium-calcineurin-NFAT signaling pathway and that this activation is important for the PTH induction of *COX-2* expression in osteoblasts. These results suggest that crosstalk between the cAMP/PKA pathway and the calcium-calcineurin-NFAT pathway may play a role in other functions of PTH that are important for the regulation of bone turnover.
